# Persulfidation proteome reveals the regulation of protein function by hydrogen sulfide in diverse biological processes in Arabidopsis

**DOI:** 10.1093/jxb/erx294

**Published:** 2017-08-24

**Authors:** Angeles Aroca, Juan M Benito, Cecilia Gotor, Luis C Romero

**Affiliations:** 1 Instituto de Bioquímica Vegetal y Fotosíntesis; 2 Instituto de Investigaciones Química, Consejo Superior de Investigaciones Científicas y Universidad de Sevilla, Avenida Américo Vespucio, Sevilla, Spain

**Keywords:** Cysteine, hydrogen sulfide, mass spectrometry, persulfidation, post-translational modification, proteomics

## Abstract

Hydrogen sulfide-mediated signaling pathways regulate many physiological and pathophysiological processes in mammalian and plant systems. The molecular mechanism by which hydrogen sulfide exerts its action involves the post-translational modification of cysteine residues to form a persulfidated thiol motif, a process called protein persulfidation. We have developed a comparative and quantitative proteomic analysis approach for the detection of endogenous persulfidated proteins in wild-type Arabidopsis and *L-CYSTEINE DESULFHYDRASE 1* mutant leaves using the tag-switch method. The 2015 identified persulfidated proteins were isolated from plants grown under controlled conditions, and therefore, at least 5% of the entire Arabidopsis proteome may undergo persulfidation under baseline conditions. Bioinformatic analysis revealed that persulfidated cysteines participate in a wide range of biological functions, regulating important processes such as carbon metabolism, plant responses to abiotic and biotic stresses, plant growth and development, and RNA translation. Quantitative analysis in both genetic backgrounds reveals that protein persulfidation is mainly involved in primary metabolic pathways such as the tricarboxylic acid cycle, glycolysis, and the Calvin cycle, suggesting that this protein modification is a new regulatory component in these pathways.

## Teaser

Protein persulfidation in the Arabidopsis proteome is widespread and is the molecular mechanism by which hydrogen sulfide performs its signaling role.

## Introduction

Hydrogen sulfide (H_2_S) has been referred to as the third gasotransmitter in animal and plant cells and is as important as nitric oxide (NO), carbon monoxide (CO), and hydrogen peroxide (H_2_O_2_) (García-Mata and Lamattina, 2010; Vandiver and Snyder, 2012; Kimura, 2014). These small molecules possess high permeability that allows them to cross biological membranes and to act as signaling molecules. H_2_S dissociates easily under physiological conditions. It is therefore assumed that hydrogen sulfide pools include H_2_S, HS^-^, and S^2-^, but the active form of hydrogen sulfide in cells has not been fully clarified. H_2_S is involved in many physiological and pathological processes in animals, including cell proliferation, apoptosis, inflammatory processes, hypoxia protection, neuromodulation, and cardioprotection, as previously described (Wang, 2014; Olas, 2015; Paul and Snyder, 2015*b*).

More recently, regulatory properties of H_2_S have emerged in plants, with protective effects reported against oxidative and metal stresses (Zhang *et al.*, 2008; Wang *et al.*, 2010; Zhang *et al.*, 2010; Li *et al.*, 2012*a*; Shen *et al.*, 2013; Sun *et al.*, 2013; Fang *et al.*, 2016;), drought and heat tolerance (Li *et al.*, 2012*b*; Shen *et al.*, 2013), and osmotic and saline stresses (Shi *et al.*, 2013). H_2_S is involved in regulating important physiological processes in plants, such as stomatal closure/aperture (García-Mata and Lamattina, 2010; Lisjak *et al.*, 2010; Jin *et al.*, 2013; Scuffi *et al.*, 2014; Papanatsiou *et al.*, 2015), the modulation of photosynthesis (Chen *et al.*, 2011), and autophagy regulation (Alvarez et al., 2012*b*; Gotor *et al.*, 2013; Romero *et al.*, 2014; Laureano-Marin *et al.*, 2016).

In mammals, endogenous H_2_S is produced by the action of two pyridoxal phosphate (PLP)–dependent enzymes, cystathionine γ-lyase (CSE) and cystathionine β-synthase (CBS), and the PLP-independent enzyme 3-mercaptopyruvate sulfurtransferase (3MST), as steps in the cysteine catabolic pathway (Kimura, 2015). In plant systems, the bulk of sulfide production occurs in chloroplasts through the photosynthetic sulfate-assimilation pathway, in which sulfate is reduced to sulfide and assimilated into cysteine (Takahashi *et al.*, 2011; Garcia *et al.*, 2015). In mitochondria, the enzyme CYANOALANINE SYNTHASE C1 (CAS-C1) also generates sulfide through the catalysis of cysteine and cyanide to form β-cyanoalanine (Yamaguchi *et al.*, 2000; Alvarez *et al*., 2012*c*). However, the sulfide produced in these organelles is dissociated into its ionized forms and therefore is unable to be transported across the membrane into the cytosol. Although several enzymatic reactions have been described but have not been characterized in detail (Riemenschneider *et al.*, 2005; Van Hoewyk *et al.*, 2008; Jin *et al.*, 2013), the production of endogenous cytosolic H_2_S has been demonstrated by L-CYSTEINE DESULFHYDRASE 1 (DES1). This enzyme catalyzes the desulfuration of cysteine to sulfide, ammonia, and pyruvate (Alvarez *et al.*, 2010; Gotor *et al.*, 2010; Alvarez *et al.*, 2012*b*). In plants, DES1 is primarily responsible for sulfide production in the cytosol and this sulfide and other sulfurating species such as sulfane sulfur or polysulfides are essential for signaling (Romero *et al.*, 2013*b*; Gotor *et al.*, 2015).

Despite numerous reports highlighting the importance of H_2_S as a signaling molecule, its mechanism of action is not yet fully understood. However, the primary signaling mechanism of H_2_S occurs through the persulfidation of reactive cysteine residues on target proteins via conversion of the thiol group (-SH) into a persulfide group (-SSH). This posttranslational modification of target proteins results in functional changes in enzymatic structures and activities, including those of Arabidopsis ascorbate peroxidase, glyceraldehyde-3-phosphate dehydrogenase, and glutamine synthetase (Aroca *et al.*, 2015), and in subcellular localizations (Mustafa *et al.*, 2009; Kimura, 2015; Paul and Snyder, 2015*c*; Aroca *et al.*, 2017). Persulfidation, previously known as S-sulfhydration, typically increases the reactivity of the modified cysteine due to the increased nucleophilicity of persulfide compared with the thiol group (Paul and Snyder, 2012; Cuevasanta *et al.*, 2015). Furthermore, quantitative data have indicated the widespread nature of persulfides in animal and plant cells (Mustafa *et al.*, 2009; Ida *et al.*, 2014). This reinforces the importance of persulfidation as a signaling process and explains the increasing interest in understanding its underlying mechanism.

A growing number of persulfidated proteins have been described using different tagging methods and proteomics approaches, including the elution of alkylated persulfides by reducing agent methods (Gao *et al.*, 2015; Longen *et al.*, 2016). Using the first described method, the modified biotin switch assay, a large number of persulfidated proteins were identified in animal and plant systems (Mustafa *et al.*, 2009; Aroca *et al.*, 2015). Nevertheless, the specificity of the blocking reagent S-methyl-methanothiosulfonate (MMTS) has been questioned by several authors. Thus, a new approach to detect persulfidated proteins was recently described, the tag-switch method (Zhang *et al.*, 2014). This method employs methylsulfonylbenzothiazole (MSBT) to block both thiols and persulfide groups in the first step; then, the disulfide bonds in persulfide adducts possess enhanced reactivity to nucleophilic attack by the cyanoacetate-based reagent CN-biotin, while thiol adducts are thioethers that do not react with nucleophiles.

Due to the growing importance of sulfide as a signaling molecule and considering our scarce knowledge regarding the functions and targets of persulfidation in plants, in this paper we performed a large-scale proteomics study of endogenously persulfidated proteins in wild-type Arabidopsis and DES1-defective (*des1*) mutant plants using the tag-switch method to shed light on the role of persulfidation and provide targets for further studies of this post-translational modification in plants.

## Materials and methods

### Plant material and growth conditions

Arabidopsis (*Arabidopsis thaliana*) wild-type ecotype Col-0 and the *des1* T-DNA insertion mutant (*des1-1*; SALK_103855) were grown in soil under a photoperiod of 16 h of white light (120 μE m^-2^ s^-1^) at 20°C and 8 h of dark at 18°C (Bermudez *et al.*, 2012).

### Tag-switch method

The biotinylation of endogenously persulfidated proteins from 30-day-old Arabidopsis plants was performed by the tag-switch method as previously described (Zhang *et al.*, 2014).

Plant leaf material weighing 1 g was ground in a mortar under liquid nitrogen and an enriched cytosolic extract was obtained following a previously described adapted method (Giavalisco *et al.*, 2003). Ground material was homogenized in a solution containing 0.125 parts (v/w) of buffer I [50 mM Tris, pH 7.1, amended with 100 mM KCl, 20% glycerol and protease inhibitor cocktail (25x) (Roche)] and 0.05 parts (v/w) of buffer II (1 mM pepstatin and 1.4 µM PMSF dissolved in ethanol) and then centrifuged at 50000 rpm for 1 h at 4ºC.

The enriched cytosolic extract was incubated with 50 mM MSBT in 50 mM Tris-HCl at pH 8, amended with 2.5% SDS, at 37°C for 30 min, followed by the addition of 20 mM CN-biotin at 37°C for 4 h. Proteins were then precipitated and pellets were washed with ice-cold acetone and resuspended in 50 mM Tris-HCl at pH 8. To purify the labeled proteins, the solution was incubated with streptavidin beads for 1 h at room temperature with frequent vortexing. The beads were intensively washed with Tris-HCl at pH 8, 600 mM NaCl, 1 mM EDTA, and 0.5% Triton X-100 and centrifuged at 3000 rpm for 5 s at room temperature. Bound proteins were eluted with a solution of 2% SDS, 30 mM biotin, 50 mM phosphate, 100 mM NaCl, 6 M urea, and 2 M thiourea for 15 min at room temperature, followed by 15 min at 96°C as previously described (Rybak *et al.*, 2004).

To determine the specificity of the method, a cytosol-enriched protein extract was obtained from the 30-day-old wild-type and *des1* Arabidopsis plants. One aliquot was incubated directly with 20 mM CN-biotin as described for tag-switch labeling, skipping the MSBT blocking step. Another aliquot was incubated with 100 mM DTT for 1 h at 37°C prior to tag-switch labeling. The proteins were then precipitated and washed, followed by incubation with MSBT and CN-biotin as described above. Samples were purified with streptavidin beads and immunoblotted using anti-HPDP-biotin (Abcam) as previously described (Aroca *et al.*, 2015).

### Identification of persulfidated proteins by LC-MS/MS analysis

To identify persulfidated proteins, tryptic digestion was performed in solution and peptide analysis was carried out by liquid chromatography and mass spectrometry analysis at the Proteomics Facility of the Centro Nacional de Biotecnología, Spain.

Protein concentration from eluted streptavidin beads was determined using a Pierce 660-nm protein assay (Thermo). An aliquot of 40 µg of protein from each treatment group was digested with sequencing-grade modified trypsin (Sigma-Aldrich) at 37°C overnight on a shaker. Three biological replicates for each condition, wild type and *des1*, were analyzed in this study.

A 1 µg aliquot of each sample was subjected to 1D-nano LC ESI-MSMS analysis using a nano liquid chromatography system (Eksigent Technologies nanoLC Ultra 1D plus, AB SCIEX) coupled to a high-speed TripleTOF 5600 mass spectrometer (AB SCIEX) with a Nanospray III source. The analytical column was a silica-based reversed-phase column C18 ChromXP, 75 µm × 15 cm, 3-µm particle size, with 120-Å pore size (Eksigent Technologies, AB SCIEX). The trap column was a C18 ChromXP (Eksigent Technologies, AB SCIEX). Peptides were separated using a 250 min gradient ranging from 2% to 90% mobile phase B (mobile phase A, 2% acetonitrile, 0.1% formic acid; mobile phase B, 100% acetonitrile, 0.1% formic acid). The injection volume was 5 µL.

Data acquisition was performed with a TripleTOF 5600 System (AB SCIEX). For IDA parameters, a 0.25 s MS survey scan in the mass range of 350–1250 m/z followed by 35 MS/MS scans of 100 ms in the mass range of 100–1800, with a total cycle time of 4 s, was performed. Switching criteria were set to ions with a mass-to-charge ratio (m/z) greater than 350 and smaller than 1250 with a charge state of 2–5 and an abundance threshold of more than 90 counts (cps). Former target ions were excluded for 20 s. The IDA rolling collision energy (CE) parameters script was used to automatically control for the CE.

MS and MS/MS data obtained for individual samples were processed using Analyst® TF 1.5.1 Software (AB SCIEX). Searches were done with an *A. thaliana* protein database from UniProt, which contains 66814 protein-coding genes and their corresponding reversed entries using the Mascot Server v. 2.5.1 (Matrix Science, London, UK). Search parameters were set as follows: acetyl (Protein N-term), CN-Biotin-Na-Sulfide (Cysteine), CN-Biotin-Sulfide (Cysteine), Methylthio (Cysteine), MSBT (Cysteine), Oxidation (Methionine), and Sulfide (Cysteine) as variable modifications. The peptide mass tolerance was set to 25 ppm and 0.05 Da for fragment masses, and two missed cleavages were allowed. False discovery rates (FDR≤1% at the PSM level) for peptide identification were manually calculated.

The mass spectrometry data have been deposited with the ProteomeXchange Consortium via the PRIDE (Vizcaino *et al.*, 2016) partner repository with the dataset identifiers PXD005168 and 10.6019/PXD005168.

Protein functional analysis and classification were performed with MapMan (Thimm *et al.*, 2004; Klie and Nikoloski, 2012). Singular enrichment analysis (SEA) of Gene Ontology (GO) was performed using the web-based tool and database AgriGO (Du *et al.*, 2010).

### Identification and quantification of persulfidated proteins by Tandem Mass Tag (TMT) Sixplex^TM^

The identification and quantification of persulfidated proteins using TMTsixplex^TM^ were performed at the same facility described above and the tryptic digestion was performed as described in the previous section.

The resulting peptides were subsequently labeled using a TMTsixplex Isobaric Mass Tagging Kit (Thermo Scientific) according to the manufacturer’s instructions, as follows: 126, wt-1; 127, *des1*-1; 128, wt-2; 129, *des1*-2; 130, wt-3; 131, *des1*-3. Three biological replicates for both conditions, wild-type and *des1*, were analyzed in this study.

A 1 µg aliquot of the labeled mixture was subjected to 1D-nano LC-electrospray ionization (ESI)-MS/MS analysis using the same methodology and equipment as in the previous section for protein identification, but in this case a 200 min gradient was used for peptide separation. Data acquisition was performed with a TripleTOF 5600 System (AB SCIEX) under the same parameters as described in the previous section. For IDA, the procedure was the same as above, except that the 0.25 s MS survey scan was followed by 30 MS/MS scans of 150 ms. Switching criteria were set the same as in the previous section, except that an abundance threshold of more than 70 counts (cps) was used. Other parameters were the same.

The mass spectrometry data have been deposited with the ProteomeXchange Consortium via the PRIDE partner repository with the dataset identifier PXD006140.

### Statistical analysis

Label-based quantification was performed using Proteobotics S.L. (Madrid, Spain). MS/MS spectra were exported to mgf format using Peak View v1.2.0.3 and searched using Mascot Server 2.5.1, OMSSA 2.1.9, X! Tandem 2013.02.01.1, X! Tandem with k-score plug-in and Myrimatch 2.2.140 against a composite target/decoy database built from the 31 551 sequences in the *A. thaliana* reference proteome at UniProt Knowledgebase, together with commonly occurring contaminants. Search engines were configured to match potential peptide candidates with a mass error tolerance of 25 ppm and fragment ion tolerance of 0.02 Da, allowing for up to two missed tryptic cleavage sites and a maximum isotope error (13C) of 1, setting Methylthio (C) as fixed modification, and TMTsixplex (N-term, K, Y), acetyl (Protein N-term), Oxidation (M), Gln->pyro-Glu (N-term Q), and Glu->pyro-Glu (N-term E) as variable modifications. An additional OMSSA search allowed for no-enzyme cleavage. CN-Biotin-Na-Sulfide (C), CN-Biotin-Sulfide (C), Sulfide (C), Methylthio (C), and MSBT (C) were incorporated as variable modifications in additional MASCOT and X! Tandem searches, in which Methylthio (C) was also considered a variable modification. Score distribution models were used to compute peptide-spectrum match p-values (Ramos-Fernandez *et al.*, 2008) and spectra recovered by a peptide-level FDR<0.01 filter were selected for quantitative analysis. Approximately 1% of the signals with the lowest quality were removed prior to further analysis. Peptides modified with CN-Biotin-Na-Sulfide (C), CN-Biotin-Sulfide (C) or Sulfide (C) were quantified separately from their parent proteins. Differential regulation was measured using linear models (Lopez-Serra *et al.*, 2014) and statistical significance was measured using q-value<0.05.

## Results

### Identification and functional classification of persulfidated proteins in wild-type Arabidopsis plants

**Fig. 1.**
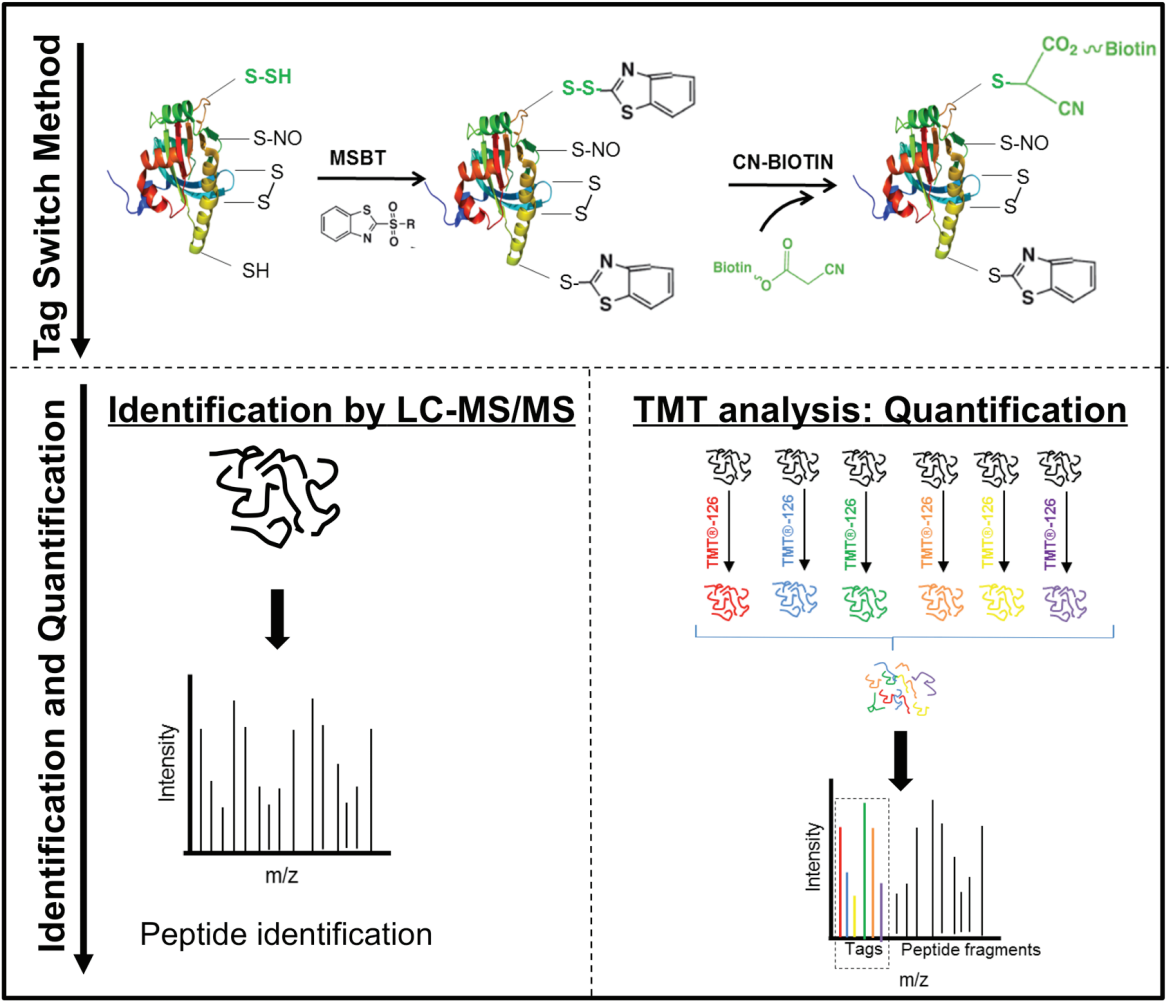
Schematic illustration of the procedure to identify and quantify persulfidated proteins in Arabidopsis leaves using the tag-switch method.

We recently reported the presence of persulfidation-modified cysteine residues in 106 proteins from Arabidopsis leaf extracts by using the modified biotin switch method (Aroca *et al.*, 2015). Recent articles have questioned the validity of this method because thiols and persulfides demonstrate similar reactivity towards MMTS (Filipovic *et al.*, 2012; Pan and Carroll, 2013). The number of Arabidopsis proteins previously reported may therefore be significantly underestimated. The publication of new methods for selective identification of persulfides on cysteine residues has motivated us to undertake a new study. The detection of endogenous persulfidated proteins in Arabidopsis leaves was performed using the tag-switch method, which labels persulfidated proteins with CN-biotin (Zhang *et al.*, 2014) (Fig. 1).

Cytosol-enriched leaf extracts from 30-day-old wild-type and *des1* mutant Arabidopsis plants grown under physiological conditions were subjected to the tag-switch method (Fig. 1). To validate the method in our plant system, protein labeling was first analyzed by performing immunoblotting using anti-biotin antibodies. Several abundant protein bands were detected in whole protein extracts labeled with the tag-switch method prior to streptavidin bead purification (see Supplementary Fig. S1A at *JXB* online). When protein extracts were treated with DTT to reduce persulfide residues prior to blocking with MSBT and CN-biotin and streptavidin-bead purification, we were unable to detect biotin-labeled proteins in the immunoblots in any wild-type or *des1* extract (Supplementary Fig. S1B). Similarly, when protein extracts were treated directly with CN-biotin without the MSBT blocking step, no signals were detected in the streptavidin-purified samples. Analogously, in the absence of the streptavidin purification step, only one biotinylated protein band was immunodetected in both wild-type and *des1* extracts previously treated with DTT (Supplementary Fig. S1B). The method was therefore considered suitable and specific for the study of persulfidation patterns in Arabidopsis in combination with a proteomics approach.

Initially, three biological replicates of CN-biotinylated proteins from Arabidopsis wild-type cytosol-enriched leaf extracts were streptavidin-purified and analyzed by LC-MS/MS. In the three biologically independent samples, we identified a total of 3147 persulfidated proteins with FDR≤1% (Supplementary Dataset S1; Comparative WT SAMPLES). Although for further analysis we have considered persulfidated proteins found in all three biological samples to be statistically significant, we cannot exclude that other proteins identified in one or two of the replicates may also be susceptible to persulfidation. Based on this criterion, we report in this work a list of 2015 persulfidated proteins in wild-type leaf extracts (Supplementary Dataset S1; WT proteins and locus). The peptides used for protein identification are provided as supplementary information (Supplementary Dataset S1). Because the protein samples analyzed in this work were isolated from the leaves of plants grown under controlled conditions with no apparent symptoms of stress, at least 5% of the Arabidopsis proteome may undergo persulfidation under baseline conditions. As a control experiment, samples with the same protein concentrations were pre-treated with DTT and processed under the same conditions as described. Following elution of the streptavidin beads, we were unable to detect persulfidated proteins via LC-MS/MS analysis in these controls.

**Fig. 2.**
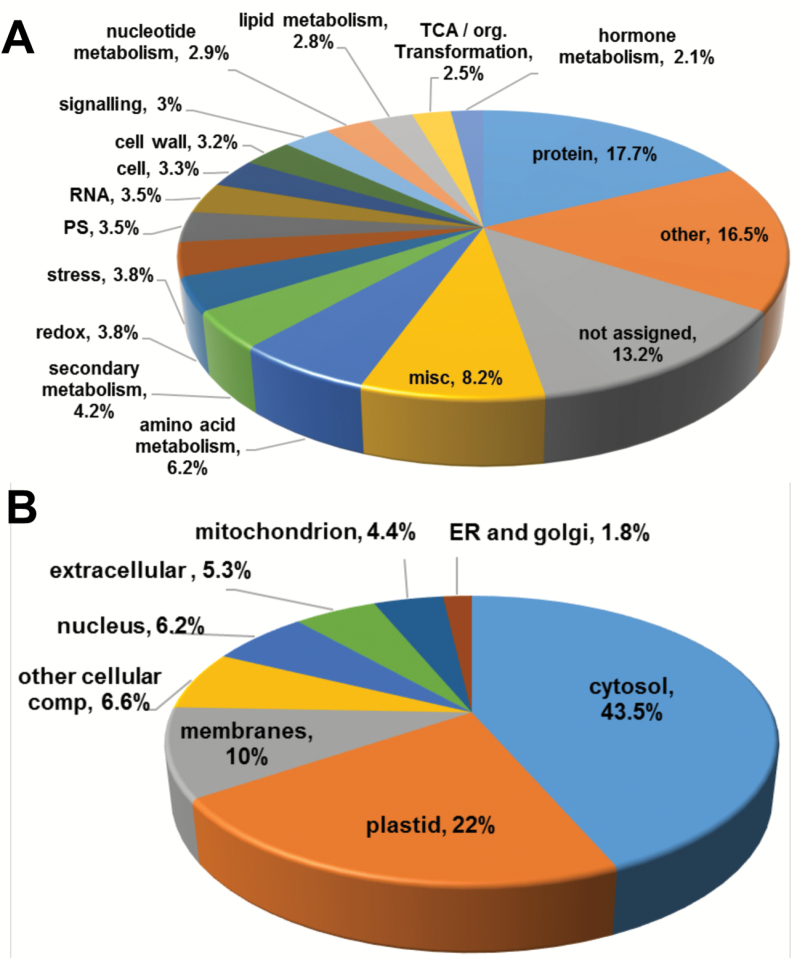
Functional classification of persulfidated proteins identified by LC-MS/MS in leaf extracts of 30-day-old Arabidopsis wild-type plants. (A) Functional classification of gene ontology (GO) terms categorized by biological processes. (B) Functional classification by subcellular localization.

The 2015 identified proteins were analyzed based on their assigned functions and classified into 34 functional groups using the MapMan nomenclature developed for plant-specific pathways and processes (Thimm *et al.*, 2004; Klie and Nikoloski, 2012) (Fig. 2A and Supplementary Table S1). The most abundant set corresponded to the general protein group, which included 17.7% of total identified proteins with 367 elements involved in protein synthesis (79 elements), subcellular targeting (25 elements), post-translational modification (38 elements), degradation (144 elements) and folding, glycosylation and assembly (38 elements). Two other important groups contained proteins involved in amino acid metabolism and miscellaneous enzyme families, representing 6.2% and 8.2% of the total, respectively (Fig. 2A), many of which are primarily involved in the regulation of primary metabolism, glycolysis, the Calvin cycle, and the tricarboxylic acid cycle. The subcellular localization of the identified proteins primarily consisted of the cytoplasm (43.5%) and plastids (22%), although proteins from the membrane and mitochondria were also highly represented (Fig. 2B).

In a previous persulfidation proteomics approach involving the biotin-switch method, 106 proteins were identified in Arabidopsis under physiological conditions (Romero *et al.*, 2013*a*; Aroca *et al.*, 2015), constituting 5% of the total newly identified persulfidated proteins. These results demonstrate a significant improvement in the method used to identify persulfidated proteins.

**Fig. 3.**
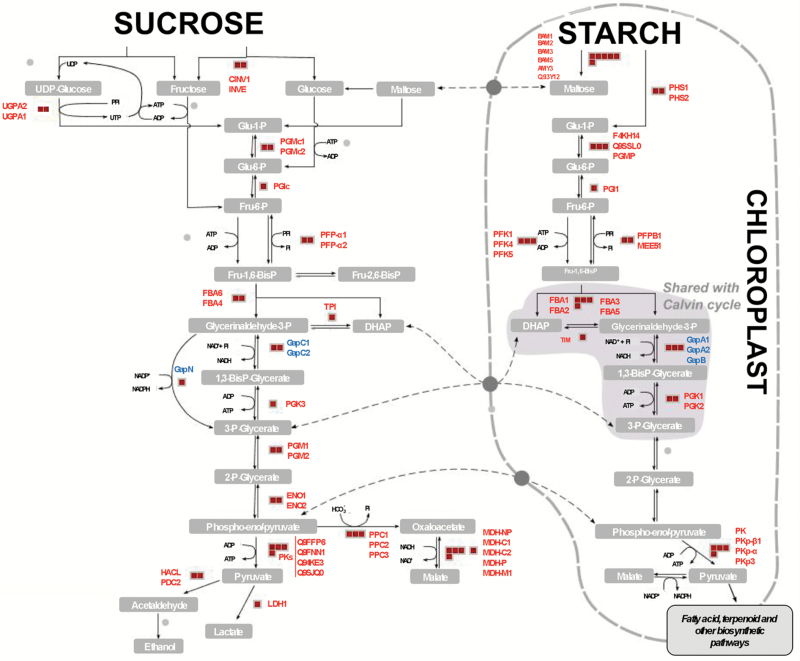
Persulfidated proteins in the plant glycolysis pathway. Red squares represent persulfidated proteins. Cytosolic, GapC1 and GapC2, chloroplastic, GapA1, GapA2, and GapB, and NADP-dependent isoforms of glyceraldehyde-3-phosphate dehydrogenase are highlighted in blue.

To study the regulatory roles and the putative functions of persulfidation in specific biological processes and to identify over-represented functional categories, we performed a singular enrichment analysis (SEA) of Gene Ontology (GO) terms using AgriGO (Du *et al.*, 2010). SEA compares each annotated gene to all annotated expressed genes. In total, 634 GO terms demonstrated significant over-representation (FDR≤0.001) (Supplementary Fig. S2). The GO enrichment analysis revealed a significant number of persulfidated proteins involved in various aspects of carbon metabolism and glycolysis; for example, 57 proteins annotated in the reference *A. thaliana* database related to the GO category ‘glycolysis’ (GO:0006096), of which 30 proteins (52.6%) were identified as persulfidated proteins, including the two cytosolic glyceraldehyde-3-phosphate dehydrogenases, GapC1 and GapC2, previously identified in Arabidopsis (Aroca *et al.*, 2015). Moreover, the regulatory function of sulfide in the nuclear localization of these proteins has been very recently demonstrated and the persulfidated cysteine residue on nuclear GapC1 identified (Aroca *et al.*, 2017). The three chloroplastic glyceraldehyde-3-phosphate dehydrogenase forms, GapA1, GapA2, and GapB, and the NADP-dependent isoforms, GapN, were also identified in the GO category. Other prominent proteins involved in glycolysis were identified as persulfidated, including several phosphofructokinases and enolases (Fig. 3 and Supplementary Table S2). An enriched subset of proteins involved in ‘tRNA aminoacylation’ (GO:0006418) was also identified, highlighting the importance of persulfidation in protein translation. Thus, almost half, 41 out of 84, of the total tRNA aminoacylation proteins represented in the reference database were identified as persulfidated proteins (Supplementary Table S3). Two other enriched categories included proteins involved in ‘jasmonic acid biosynthesis’ (GO:0009695), with 11 proteins (37.9%) out of the 29 annotated in Arabidopsis, including 12-oxophytodienoate reductases 1 and 3 and lipoxygenase 2 (Supplementary Table S4), and ‘abiotic stress responses’ (GO:0009628), comprising 214 proteins (14.5%) out of 1471 (Supplementary Table S5). Thus, the GO categorization of persulfidated proteins revealed the involvement of a large number of these proteins in regulating important biological processes, such as carbon metabolism, plant responses to abiotic stresses, plant growth and development, and RNA translation.

### Identification of persulfidated proteins in the *des1* mutant

Although the Arabidopsis genome contains several genes coding for putative D- and L-cysteine desulfhydrases that produce sulfide (Jin and Pei, 2015), only the protein DES1 has been extensively characterized *in vitro* and *in vivo*. The loss of protein function and reduced sulfide production in the mutant *des1* are related to plant physiological processes, such as senescence, autophagy, stomatal closure, and immunity (Alvarez *et al.*, 2010; Alvarez et al., 2012*a*; Alvarez et al., 2012*b*; Scuffi *et al.*, 2014; Laureano-Marin et al., 2016). To determine whether the synthesis of sulfide catalyzed by DES1 acts on cells through the protein persulfidation mechanism, we analyzed the basal levels of persulfidation in cytosol-enriched *des1* leaf extracts following the same methodology previously utilized with wild-type plants (Fig. 1). Although we used *des1* leaves from 30-day-old plants for comparison purposes, the mutation in *DES1* causes accelerated plant growth, premature leaf senescence, and altered transcriptional levels compared with wild-type plants (Alvarez *et al.*, 2012*b*).

Three biological replicates of CN-biotinylated proteins were analyzed under the same conditions and protocols as those employed for wild-type plants. A total of 3242 proteins were identified in the three samples (Supplementary Dataset S2; Comparative DES SAMPLES); 85% of these proteins were also identified in wild-type plants. Following the same criteria implemented for wild-type plants, 2130 proteins were identified in all three samples and, therefore, considered to be statistically significant as persulfidated proteins in the *des1* mutant (Supplementary Dataset S2; DES1 Proteins and locus). Functional classification of the 2130 proteins based on MapMan ontology showed no significant differences compared with wild-type (Supplementary Fig. S3).

A qualitative comparison between the lists of identified persulfidated proteins in the wild-type and *des1* lines was performed based on our criterion of being present in all three biological replicates. The comparison showed that 184 proteins were predominantly found in wild-type plants and 299 proteins in the *des1* mutant, suggesting the ability of sulfurating species generated in the cells in each genetic background to modify specific targets (Supplementary Table S6 and Datasets S1 and S2).

### Quantitative comparison of persulfidation patterns in wild-type and *des1* plants

To better understand the role of DES1 in sulfide-mediated persulfidation, a quantitative approach was performed using 6-plex tandem mass tag (TMT) isobaric peptide labeling. For this purpose, the same samples used for wild-type (three replicate samples) and *des1* (three replicate samples) LC-MS/MS analysis were labeled with a set of six isobaric compounds containing different numbers of heavy isotopes in the mass reporter region, which result in unique reporter masses during tandem MS/MS for sample identification and relative quantitation (Fig. 1).

**Fig. 4.**
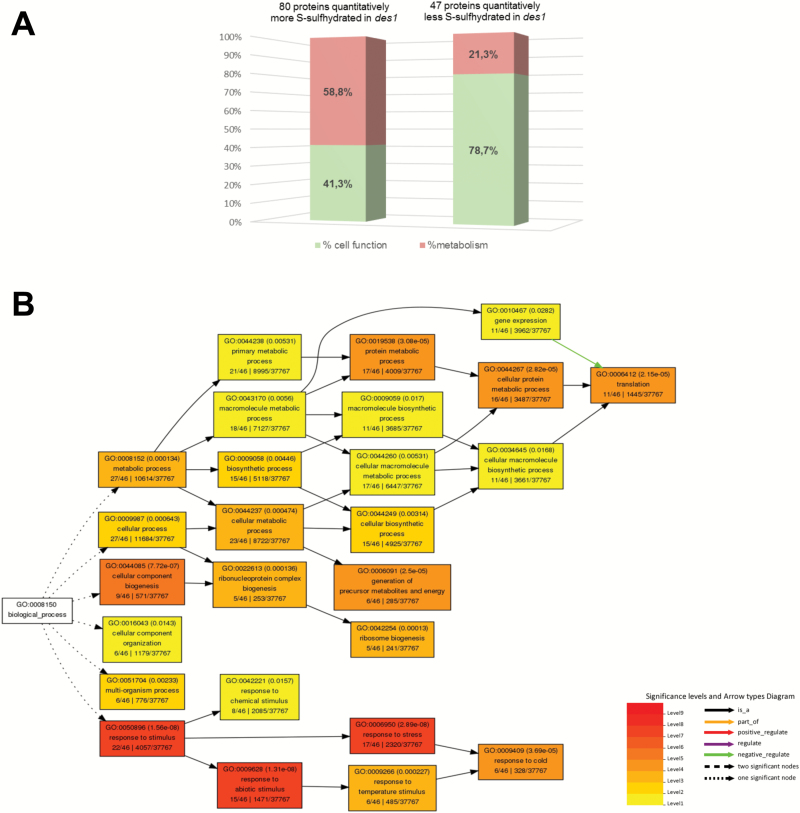
Functional characterization of proteins quantitatively regulated by persulfidation. (A) Functional distribution of proteins differentially regulated by persulfidation in *des1* and wild-type plants. (B) Singular enrichment analysis performed with AgriGO to identify enriched gene ontologies associated with proteins negatively regulated by persulfidation in *des1* mutants in comparison with persulfidation patterns in wild-type plants. Box colors indicate levels of statistical significance: yellow, 0.05; orange, e−5; red, e−9.

TMTsixplex identified 1937 proteins based on 41213 unique peptides with a FDR≤1% (Supplementary Dataset S3). The quantification of proteins found in the *des1* samples revealed a significantly different representation of 127 persulfidated proteins compared with wild-type, with 80 proteins being more abundant and 47 proteins being less abundant, with a q-value≤0.05 (Supplementary Table S7 and Dataset S3). MapMan ontology indicated the involvement of 58.8% of these over-represented proteins in primary metabolism routes, such as the tricarboxylic acid cycle, glycolysis, and the Calvin cycle. However, 78.7% of the under-represented proteins in *des1* also took part in different cell functions, such as protein synthesis or degradation, biotic and abiotic stress responses, the redox response, and calcium signaling, while only 21.3% of these proteins participated in primary metabolism pathways (Fig. 4A). A singular enrichment analysis of gene ontology was performed using the agriGO web-based toolkit to analyze the 47 proteins associated with higher levels of persulfidation in wild-type plants than in the *des1* mutant. A large number of these proteins were identified in translation, 11 proteins out of 46 (GO:0006412), and response to stress, 17 proteins out of 46 (GO:0006950) (Fig. 4B).

### Comparison analysis of persulfidation and nitrosylation proteomes

**Fig. 5.**
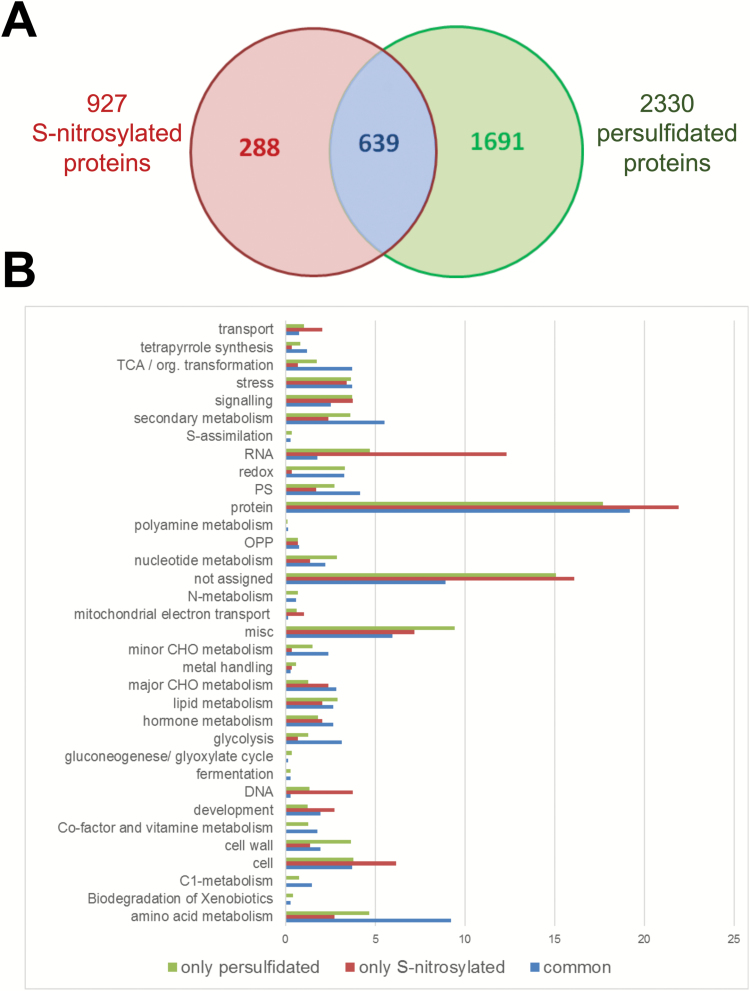
Comparison of S-nitrosylated proteins and persulfidated proteins identified in Arabidopsis plants. (A) Venn diagram of total S-nitrosylated identified proteins in wild-type and *gsnor1-3* mutant (Hu *et al.*, 2015) and of total persulfidated identified proteins in wild-type and *des1* mutant in this work. (B) Functional classification of gene ontology (GO) terms categorized by biological processes.

Although the sulfhydryl groups in protein Cys residues undergo an array of oxidative reactions and modifications, many studies have established NO-dependent S-nitrosylation as fundamental for the regulation of diverse protein activities. Since protein persulfidation and nitrosylation are similar in terms of their chemical and biological determinants, we have compared our persulfidation results with the S-nitrosylation proteome analysis previously reported. The latest and largest nitrosylation proteome of Arabidopsis has been reported by the Zuo group (Hu *et al.*, 2015), identifying 927 endogenously S-nitrosylated proteins in the *gsnor1-3* mutant that show increased levels of NO compared with wild-type. A comparison of the persulfidated and nitrosylated Arabidopsis proteomes shows that 639 proteins are susceptible to being both persulfidated and nitrosylated (Fig. 5A and Supplementary Table S8). Among others, the cytosolic glyceraldehyde-3-phosphate dehydrogenase (GAPC1) may be nitrosylated at residue Cys^156^ (Vescovi *et al.*, 2013) and persulfidated at residue Cys^160^ (Aroca *et al.*, 2017), and the functional consequences differ between modifications; persulfidation determines its nuclear localization and nitrosylation does not. However, the same GAPDH protein sites reported to undergo nitrosylation have also been found to undergo persulfidation in mammalian tissue (Hara *et al.*, 2005; Mustafa *et al.*, 2009).

Functional classification of the GO terms categorized by biological processes shows that proteins that are only nitrosylated are predominantly enriched in the RNA and DNA categories despite the number of identified proteins being significantly lower (Fig. 5B).

## Discussion

### Protein persulfidation of cysteine residues is an important mechanism involved in diverse biological processes

Many physiological and pathophysiological processes are mediated by signaling pathways involving H_2_S in mammalian and plant systems (Gotor *et al.*, 2013; Romero *et al.*, 2014; Jin and Pei, 2015; Kimura, 2015; Paul and Snyder, 2015*a*). The molecular mechanism by which H_2_S exerts its action has been identified and involves the defined oxidative post-translational modification of cysteine residues to form a persulfidated thiol motif, a process called protein persulfidation (Mustafa *et al.*, 2009; Mishanina *et al.*, 2015; Paul and Snyder, 2015*c*). One of the major challenges in the last few years has been the development of detection methods specific to protein persulfidation because one critical step is to differentiate cysteine persulfide residues from cysteine thiols. The tag-switch method, which directly labels proteins with persulfidated residues by forming stable thioethers, has allowed us to significantly increase the number of identified proteins that undergo this modification in Arabidopsis leaf extracts from 106, identified using the modified biotin-switch method (Aroca *et al.*, 2015), to more than 2000 proteins present in all the three biological replicates in this work. In the traditional method, the SH-blocking reagent MMTS does not adequately distinguish between cysteine persulfide (-SSH) and cysteine thiols (-SH) as reported (Mustafa *et al.*, 2009) because both demonstrate similar reactivity (Pan and Carroll, 2013). The reaction with biotin-HPDP to form biotin-labeled proteins is therefore under-represented. The tag-switch method detected almost 4000 proteins in samples extracted from mature leaves; however, for further protein analysis, we took a more restrictive approach in which we considered only those proteins detected in all three biological samples to be validated. This method was previously developed in mammalian systems to investigate recombinant proteins, such as bovine serum albumin (BSA) and glyceraldehyde-3-phophate dehydrogenase (GAPDH), followed by testing to detect intracellular protein persulfides in cell extracts, protein immunoprecipitation, and fluorescence microscopy (Zhang *et al.*, 2014; Park *et al.*, 2015; Wedmann *et al.*, 2016). We demonstrated the specificity of the tag-switch method on Arabidopsis protein extracts by treating the protein samples with DTT to chemically reduce cysteine persulfide residues to thiols prior to the procedure. Under these conditions, we were not able to detect labeled proteins by protein blotting or even by LC-MS/MS after streptavidin-bead purification.

The 2015 persulfidated proteins identified in wild-type plants represent at least 5% of the entire proteome of 35386 proteins encoded by the *A. thaliana* genome. However, this number may be even higher because although the tag-switch method involves enrichment for protein identification, very low abundance proteins may be lost during the procedure. In addition, we have used the stringent criterion of being identified in all three replicates but we cannot exclude that those proteins identified in one or two replicates are also persulfidated at a lower abundance and thus went undetected. Our data suggest the widespread distribution of this post-translational modification in the plant proteome and that persulfidation may have the same level of impact as phosphorylation or glycosylation modifications. Moreover, our proteomics analysis was performed on plants grown under physiological conditions and the magnitude of this modification may be even higher under conditions where sulfide has been demonstrated to play a signaling role, such as plant responses to a variety of plant stresses (Zhang *et al.*, 2008; Wang *et al.*, 2010; Li *et al.*, 2012b; Shen *et al.*, 2013; Shi *et al.*, 2013), autophagy (Alvarez et al., 2012*b*; Gotor *et al.*, 2013; Romero *et al.*, 2014; Laureano-Marin *et al.*, 2016), stomatal movement (Garcia-Mata and Lamattina, 2010; Lisjak *et al.*, 2010; Jin *et al.*, 2013; Scuffi *et al.*, 2014; Papanatsiou *et al.*, 2015) and photosynthesis (Chen *et al.*, 2011). Another factor that we are unable to exclude is changes in persulfidation patterns under specific conditions; thus, performing the described method on protein extracts from Arabidopsis plants subjected to any of these specific conditions is of major interest.

The GO classification of proteins with persulfidation-modified cysteine residues indicates their involvement in a large range of biological processes. The majority of the identified proteins are located in the cytosol (46%) and the chloroplast (25%), which are compartments with sulfide concentrations calculated to be approximately 55 µM and 125 µM, respectively, in Arabidopsis (Krueger *et al.*, 2009). It has been estimated that chloroplasts contain between 2000 and 3500 proteins, approximately 10% of all predicted protein-encoded genes (van Wijk and Baginsky, 2011); thus, protein persulfidation may be highly significant in this compartment. Indeed, hydrogen sulfide has been described to enhance photosynthesis in spinach seedlings by, among other effects, increasing the activity of the ribulose-1,5-bisphosphate carboxylase (Rubisco) enzyme (Chen *et al.*, 2011). One of the largest groups identified in this work consists of proteins involved in carbon assimilation, energy metabolism, and glycolytic flux. Several enzymes from the Calvin-Benson cycle are also present in our analysis, such as the Rubisco large and small subunits, the Rubisco activase, the chloroplastic glyceraldehyde-3-phosphate dehydrogenase, phosphoribulokinase (PRK), fructose-1,6-bisphosphatase (FBPase), sedoheptulose-1,7-bisphosphatase (SBPase), and transketolase. Regulation of Calvin–Benson cycle enzymes based on redox post-translational modiﬁcations was recognized very early in plants and involves different states of cysteine residue oxidation (Buchanan and Balmer, 2005), now also including persulfidation. These enzymes exhibit low activity in the dark, with oxidized cysteine residues forming disulfides, and are activated in the light, with reduced cysteine residues. All of these enzymes are regulated by the ferredoxin/thioredoxin (Fd/TRX) system, which plays a crucial role in redox- and light-dependent reactions in chloroplasts through the reduction of regulatory disulfides (Buchanan and Balmer, 2005).). Clearly, there is a substantial interconnection between photosynthesis and the source of hydrogen sulfide in chloroplasts because the primary sulfate assimilation pathway in photosynthetic organisms occurs in the chloroplast, where sulfate ions are reduced to sulfide in the light (Schmidt and Trebst, 1969). Sulfide *per se* is not the sulfurating species but rather polysulfides generated via the mild oxidation of sulfide (Kimura, 2015; Mishanina *et al.*, 2015). Post-translational modiﬁcation via persulfidation may be an intermediate regulatory step for the enzymes of the Calvin–Benson cycle between inhibition in the dark, via the oxidation of cysteines to disulfides, and activated enzyme activity under illumination via reduction to thiol. The persulfidation of cysteine residues is reverted *in vitro* by thioredoxins and glutaredoxin/glutathione reductase/glutathione systems as demonstrated for persulfidated mammalian PROTEIN TYROSINE PHOSPHATASE 1B (PTP1B) and BSA (Krishnan *et al.*, 2011; Doka *et al.*, 2016; Wedmann *et al.*, 2016). Persulfidated proteins in the chloroplast could therefore also be reduced and regulated by the chloroplastic thioredoxin machinery.

Sulfate and nitrate assimilation are dependent upon and interact with carbon assimilation because they are regulated by the supply of carbon skeletons produced during CO_2_ assimilation (Oaks, 1994; Kopriva *et al.*, 2002). Furthermore, the regulatory interaction between the sulfate and nitrate assimilation pathways is well established as a deficiency in one element represses the other pathway (Takahashi *et al.*, 2011). Levels of O-acetylserine, the carbon-nitrogen molecule into which sulfide is incorporated to form cysteine, connect these pathways. Curiously, the major enzymes involved in nitrogen assimilation are susceptible to persulfidation, such as nitrate reductase, NIA1 and NIA2, nitrite reductase, NIR1, glutamine synthetase, GS1, and ferredoxin-dependent glutamate synthase, GLU1(this work and Aroca *et al.*, 2015). We previously characterized the regulation of glutamine synthetase by hydrogen sulfide via the inhibition of its activity; this process is reversed by reduction with dithiothreitol (Aroca *et al.*, 2015). Enzyme regulation by persulfidation in the nitrogen-assimilation pathway therefore represents another step in the molecular mechanism underlying the coordination of both routes.

In addition to playing a regulatory role in primary carbon and nitrogen assimilation, H_2_S is also essential for the degradation and recycling of cellular components in plant cells induced by carbon or nitrogen starvation, regulating the mechanism of autophagy and acting as a repressor of this process under normal growth conditions and sufficient nutrient availability (Alvarez et al., 2012*b*; Laureano-Marin *et al.*, 2016). In *des1* mutants exhibiting a reduced cytosolic H_2_S repressor signal, autophagy is therefore induced under sufficient nutrient availability via an unknown signaling mechanism. Proteomics analysis performed on wild-type samples revealed the susceptibility of the autophagy (ATG)-related proteins, ATG18a, ATG3, ATG5, and ATG7, to persulfidation, although the three last proteins were identified in only one replicate. In the proteomics analysis performed in *des1* mutants, we detected ATG18a, but no other ATG members, in the full protein set, suggesting that persulfidation may be the molecular mechanism through which sulfide regulates autophagy in plant cells. However, the identification of autophagy-related molecular protein targets requires further specific studies.

Similar to the results obtained in this work, proteomics analysis of pancreatic ß cells recently revealed a subset of proteins with induced persulfidation involved in aminoacyl-tRNA biosynthesis (Gao *et al.*, 2015). Nearly half of the proteins involved in the synthesis of aminoacyl-tRNA have been identified as persulfidated in wild-type Arabidopsis samples. Efficient protein translation depends on the availability of aminoacyl-tRNA and the ratio of correctly acylated to misacylated tRNA. Although there is little information regarding the redox regulation of these enzymes, the sulfenylation of cysteine residues in aminoacyl-tRNA synthases in *Escherichia coli* during oxidative stress increases misacylation and leads to error-prone translation (Ling and Soll, 2010). As hydropersulfides do not form in a direct reaction between H_2_S and protein –SH, the persulfidation reaction occurs when one of the cysteine residues in the target protein, found in the oxidized form, is subsequently modified to a persulfide moiety. Additional control of translation by thiolation was reported in yeast, in which intracellular methionine and cysteine availability directly control the thiolation status of wobble-uridine (U_34_) nucleotides present on lysine, glutamine, and glutamate tRNAs to regulate cellular translational capacity and metabolic homeostasis (Laxman *et al.*, 2013). Cellular sulfur content therefore plays a fundamental role in the translational machinery of cells.

### Sulfide generated by DES1 has specific regulatory roles

A quantitative comparison between wild-type and *des1* plants revealed small but significant differences in protein persulfidation profiles. Although the majority of proteins carrying persulfidated residues are the same in wild-type and *des1* plants, some of these proteins slightly but significantly change their persulfidation levels as determined by quantitative proteomics analysis. In addition, wild-type and *des1* plants also demonstrated specific persulfidated protein targets under our experimental conditions. Although the *des1* mutant is defective for the cytosolic H_2_S-generating enzyme DES1, DES1 is not the only cysteine-dependent sulfide producer present in the cell and therefore *des1* mutants retain the capacity to generate sulfide via both photosynthetic sulfate reduction and the actions of other cysteine desulfhydrase enzymes. The *des1* mutant demonstrates two clearly distinct characteristics compared with wild-type plants that affect protein persulfidation in the mutant plants. First, the *des1* mutant contains 30% less sulfur under steady-state growth conditions, primarily in the cytosol; second, it contains higher cysteine levels that may locally promote the production of oxidative species by driving the Fenton reaction (Park and Imlay, 2003; Alvarez *et al.*, 2010).

Several proteins whose persulfidation levels are altered in *des1* plants are involved in intracellular signaling processes, including many protein kinases, phosphatases, defense response molecules, and components of the proteasome complex. Among the kinase targets, we detected SNF1-RELATED PROTEIN KINASE 2.2 (SNRK2.2) and 2.6 (OST1), which play essential roles in the abscisic acid (ABA)-dependent regulation of stomatal movement. In addition, we also detected persulfidation of the abscisic acid receptors PYRABACTIN RESISTANCE 1 (PYR1) and PYR1-LIKE PROTEIN 1 (PYL1) in both in wild-type and *des1* samples (Miyakawa *et al.*, 2013). The participation of H_2_S in stomatal closure has been widely reported (Garcia-Mata and Lamattina, 2010; Papanatsiou *et al.*, 2015) and ABA does not induce stomatal closure in isolated epidermal strips of *des1* mutants; however, this effect is restored via the application of exogenous H_2_S. Furthermore, H_2_S acts upstream of NO to regulate stomatal closure because NO depletion blocks H_2_S-dependent effects (Scuffi *et al.*, 2014). ABA binds to PYR/PYL/RCAR receptors and this complex promotes the phosphorylation and activation of SNRK2, which phosphorylates numerous downstream protein targets involved in the ABA response. OST1/SNRK2 is S-nitrosylated by NO at Cys^137^, a residue adjacent to the kinase catalytic site, provoking the inhibition of its phosphorylating activity (Wang *et al.*, 2015). As persulfidation and nitrosylation influence protein function in an opposing manner, similar to ascorbate peroxidase and GAPDH (Aroca *et al.*, 2015), OST1 is a candidate protein to modulate the cross-talk of these two gasotransmitters.

Comparison of the persulfidated proteome with a reported nitrosylated proteome shows that many of the identified proteins are susceptible to both modifications. It is interesting that in wild-type plants, with a significantly lower level of NO content, the differences in protein nitrosylation compared with the *gsnor1-3* mutant were not important, like in wild-type and *des1* plants at the level of persulfidation. These observations suggest that the production of intracellular NO and H_2_S is not enough to induce protein nitrosylation or persulfidation, respectively; therefore, additional events are necessary to generate and regulate protein modifications.

In conclusion, the tag-switch method, which is more specific and selective than the methods previously published, has allowed us to significantly increase the number of identified proteins that may be susceptible to persulfidation. Functional analysis of these proteins reveals the impact that regulation by hydrogen sulfide can exert on metabolism and cellular regulation in plants. The fact that a high number of proteins are persulfidated in both wild-type and *des1* mutant lines indicates that the basal level of persulfidation is high under normal growth conditions and that in addition to DES1, there are other enzymes or processes that can generate sulfurating species as they exist in mammalian systems. Further proteomic analyses under conditions of biotic or abiotic environmental stress and at different stages of development will shed more light on the regulatory role of sulfide in plant cells.

## Supplementary Data

Supplementary data are available at *JXB* online.

Fig. S1. Validation of the tag-switch method in wild-type and *des1* mutant Arabidopsis leaf extracts by immunoblotting with anti-biotin antibodies.

Fig. S2. Enriched Gene Ontology analysis of the 2015 loci corresponding to the persulfidated proteins identified by the tag-switch method in wild-type plants.

Fig. S3. Functional classification of persulfidated proteins identified by LC-MS/MS in the leaf extracts of 30-day-old Arabidopsis thaliana *des1* mutant plants.

Table S1. Ontology classification of persulfidated proteins in wild-type samples according to MapMan.

Table S2. Singular enrichment analysis of Gene Ontology (GO) terms related to the category “glycolysis”.

Table S3. Singular enrichment analysis of Gene Ontology (GO) terms related to the category “tRNA aminoacylation for protein translation”.

Table S4. Singular enrichment analysis of Gene Ontology (GO) terms related to the category “jasmonic acid biosynthesis”.

Table S5. Singular enrichment analysis of Gene Ontology (GO) terms related to the category “abiotic stress responses”.

Table S6. Proteins differentially labeled persulfidated in wild-type and *des1* samples.

Table S7. Quantitative comparison of persulfidation patterns in wild-type and *des1* plants.

Table S8. Commonly identified proteins in the persulfidation and nitrosylation proteome analysis.

Dataset S1. List of identified proteins in wild-type (WT) samples (FDR<1%).

Dataset S2. List of identified proteins in *des1* samples (FDR<1%).

Dataset S3. Quantitative comparison of persulfidation patterns in wild-type and *des1* plants (FDR<1%).

## Data Deposition

Mass spectrometry data. PRIDE Archive, ProteomeXchange Consortium. Dataset identifiers PXD005168, 10.6019/PXD005168, and PXD006140. 

https://www.ebi.ac.uk/pride/archive/projects/PXD005168

https://www.ebi.ac.uk/pride/archive/projects/ PXD006140

## Supplementary Material

supplementary_table_S1

supplementary_table_S2

supplementary_table_S3

supplementary_table_S4

supplementary_table_S5

supplementary_table_S6

supplementary_table_S7

supplementary_table_S8

supplementary_data_S1

supplementary_data_S2

supplementary_data_S3

supplementary_figures_S1_S3

## Abbreviations:

ABA: abscisic acid
DES1: L-CYSTEINE DESULFHYDRASE 1
FDR: false discovery rate
MMTS: S-methyl-methanothiosulfonate
MSBT: methylsulfonylbenzothiazole.

## References

[CIT0001] Álvarez C, Bermúdez MÁ, Romero LC, Gotor C, García I 2012a Cysteine homeostasis plays an essential role in plant immunity. New Phytologist 193, 165–177. 21988475 10.1111/j.1469-8137.2011.03889.x

[CIT0002] Alvarez C, Calo L, Romero LC, García I, Gotor C 2010 An O-acetylserine(thiol)lyase homolog with L-cysteine desulfhydrase activity regulates cysteine homeostasis in Arabidopsis. Plant Physiology 152, 656–669. 19955263 10.1104/pp.109.147975 PMC2815857

[CIT0003] Álvarez C, García I, Moreno I, Pérez-Pérez ME, Crespo JL, Romero LC, Gotor C 2012b Cysteine-generated sulfide in the cytosol negatively regulates autophagy and modulates the transcriptional profile in Arabidopsis. The Plant Cell 24, 4621–4634. 23144183 10.1105/tpc.112.105403 PMC3531856

[CIT0004] Álvarez C, García I, Romero LC, Gotor C 2012c Mitochondrial sulfide detoxification requires a functional isoform O-acetylserine(thiol)lyase C in Arabidopsis thaliana. Molecular Plant 5, 1217–1226. 22511607 10.1093/mp/sss043

[CIT0005] Aroca A, Benito JM, Gotor C, Romero LC 2017 Data from: Persulfidation proteome reveals the regulation of protein function by hydrogen sulfide in diverse biological processes in Arabidopsis PRIDE Archive, ProteomeXchange Consortium https://www.ebi.ac.uk/pride/archive/projects/PXD005168 PXD006140https://www.ebi.ac.uk/pride/archive/projects/

[CIT0006] Aroca A, Schneider M, Scheibe R, Gotor C, Romero LC 2017 Hydrogen sulfide regulates the cytosolic/nuclear partitioning of glyceraldehyde-3-phosphate dehydrogenase by enhancing its nuclear localization. Plant & Cell Physiology 58, 983–992. 28444344 10.1093/pcp/pcx056

[CIT0007] Aroca Á, Serna A, Gotor C, Romero LC 2015 S-sulfhydration: a cysteine posttranslational modification in plant systems. Plant Physiology 168, 334–342. 25810097 10.1104/pp.15.00009 PMC4424021

[CIT0008] Bermúdez MÁ, Galmés J, Moreno I, Mullineaux PM, Gotor C, Romero LC 2012 Photosynthetic adaptation to length of day is dependent on S-sulfocysteine synthase activity in the thylakoid lumen. Plant Physiology 160, 274–288. 22829322 10.1104/pp.112.201491 PMC3440205

[CIT0009] Buchanan BB, Balmer Y 2005 Redox regulation: a broadening horizon. Annual Review of Plant Biology 56, 187–220. 10.1146/annurev.arplant.56.032604.144246 15862094

[CIT0010] Chen J, Wu FH, Wang WH, Zheng CJ, Lin GH, Dong XJ, He JX, Pei ZM, Zheng HL 2011 Hydrogen sulphide enhances photosynthesis through promoting chloroplast biogenesis, photosynthetic enzyme expression, and thiol redox modification in Spinacia oleracea seedlings. Journal of Experimental Botany 62, 4481–4493. 21624977 10.1093/jxb/err145 PMC3170546

[CIT0011] Cuevasanta E, Lange M, Bonanata J, Coitiño EL, Ferrer-Sueta G, Filipovic MR, Alvarez B 2015 Reaction of Hydrogen Sulfide with Disulfide and Sulfenic Acid to Form the Strongly Nucleophilic Persulfide. The Journal of Biological Chemistry 290, 26866–26880. 26269587 10.1074/jbc.M115.672816 PMC4646399

[CIT0012] Dóka É, Pader I, Bíró A 2016 A novel persulfide detection method reveals protein persulfide- and polysulfide-reducing functions of thioredoxin and glutathione systems. Science Advances 2, e1500968. 26844296 10.1126/sciadv.1500968 PMC4737208

[CIT0013] Du Z, Zhou X, Ling Y, Zhang Z, Su Z 2010 agriGO: a GO analysis toolkit for the agricultural community. Nucleic Acids Research 38, W64–W70. 20435677 10.1093/nar/gkq310 PMC2896167

[CIT0014] Fang H, Liu Z, Jin Z, Zhang L, Liu D, Pei Y 2016 An emphasis of hydrogen sulfide-cysteine cycle on enhancing the tolerance to chromium stress in Arabidopsis. Environmental Pollution 213, 870–877. 27038574 10.1016/j.envpol.2016.03.035

[CIT0015] Filipovic MR, Miljkovic JLj, Nauser T, Royzen M, Klos K, Shubina T, Koppenol WH, Lippard SJ, Ivanović-Burmazović I 2012 Chemical characterization of the smallest S-nitrosothiol, HSNO; cellular cross-talk of H2S and S-nitrosothiols. Journal of the American Chemical Society 134, 12016–12027. 22741609 10.1021/ja3009693 PMC3408084

[CIT0016] Gao XH, Krokowski D, Guan BJ 2015 Quantitative H2S-mediated protein sulfhydration reveals metabolic reprogramming during the integrated stress response. Elife 4, e10067. 26595448 10.7554/eLife.10067 PMC4733038

[CIT0017] Garcia I, Gotor C, Romero LC 2015 Cysteine homeostasis. In: D’Mello JPF, ed. Amino Acids in Higher Plants. Wallingford, United Kingdom: CABI Publishing, 219–233.

[CIT0018] García-Mata C, Lamattina L 2010 Hydrogen sulphide, a novel gasotransmitter involved in guard cell signalling. New Phytologist 188, 977–984. 20831717 10.1111/j.1469-8137.2010.03465.x

[CIT0019] Giavalisco P, Nordhoff E, Lehrach H, Gobom J, Klose J 2003 Extraction of proteins from plant tissues for two-dimensional electrophoresis analysis. Electrophoresis 24, 207–216. 12652593 10.1002/elps.200390016

[CIT0020] Gotor C, Alvarez C, Bermúdez MA, Moreno I, García I, Romero LC 2010 Low abundance does not mean less importance in cysteine metabolism. Plant Signaling & Behavior 5, 1028–1030. 20699647 10.4161/psb.5.8.12296 PMC3115188

[CIT0021] Gotor C, García I, Crespo JL, Romero LC 2013 Sulfide as a signaling molecule in autophagy. Autophagy 9, 609–611. 23328265 10.4161/auto.23460 PMC3627676

[CIT0022] Gotor C, Laureano-Marín AM, Moreno I, Aroca Á, García I, Romero LC 2015 Signaling in the plant cytosol: cysteine or sulfide?Amino Acids 47, 2155–2164. 24990521 10.1007/s00726-014-1786-z

[CIT0023] Hara MR, Agrawal N, Kim SF 2005 S-nitrosylated GAPDH initiates apoptotic cell death by nuclear translocation following Siah1 binding. Nature Cell Biology 7, 665–674. 15951807 10.1038/ncb1268

[CIT0024] Hu J, Huang X, Chen L, Sun X, Lu C, Zhang L, Wang Y, Zuo J 2015 Site-specific nitrosoproteomic identification of endogenously S-nitrosylated proteins in Arabidopsis. Plant Physiology 167, 1731–1746. 25699590 10.1104/pp.15.00026 PMC4378176

[CIT0025] Ida T, Sawa T, Ihara H 2014 Reactive cysteine persulfides and S-polythiolation regulate oxidative stress and redox signaling. Proceedings of the National Academy of Sciences USA 111, 7606–7611. 10.1073/pnas.1321232111 PMC4040604 24733942

[CIT0026] Jin Z, Pei Y 2015 Physiological implications of hydrogen sulfide in plants: pleasant exploration behind its unpleasant odour. Oxidative Medicine and Cellular Longevity 2015, 397502. 26078806 10.1155/2015/397502 PMC4442293

[CIT0027] Jin Z, Xue S, Luo Y, Tian B, Fang H, Li H, Pei Y 2013 Hydrogen sulfide interacting with abscisic acid in stomatal regulation responses to drought stress in Arabidopsis. Plant Physiology and Biochemistry 62, 41–46. 23178483 10.1016/j.plaphy.2012.10.017

[CIT0028] Kimura H 2014 The physiological role of hydrogen sulfide and beyond. Nitric Oxide: Biology and Chemistry 41, 4–10. 24491257 10.1016/j.niox.2014.01.002

[CIT0029] Kimura H 2015 Signaling of hydrogen sulfide and polysulfides. Antioxidants & Redox Signaling 22, 347–349. 25178405 10.1089/ars.2014.6082 PMC4307096

[CIT0030] Klie S, Nikoloski Z 2012 The choice between mapman and gene ontology for automated gene function prediction in plant science. Frontiers in Genetics 3, 115. 22754563 10.3389/fgene.2012.00115 PMC3384976

[CIT0031] Kopriva S, Suter M, von Ballmoos P, Hesse H, Krähenbühl U, Rennenberg H, Brunold C 2002 Interaction of sulfate assimilation with carbon and nitrogen metabolism in Lemna minor. Plant Physiology 130, 1406–1413. 12428005 10.1104/pp.007773 PMC166659

[CIT0032] Krishnan N, Fu C, Pappin DJ, Tonks NK 2011 H2S-Induced sulfhydration of the phosphatase PTP1B and its role in the endoplasmic reticulum stress response. Science Signaling 4, ra86. 22169477 10.1126/scisignal.2002329 PMC3328411

[CIT0033] Krueger S, Niehl A, Lopez Martin MC 2009 Analysis of cytosolic and plastidic serine acetyltransferase mutants and subcellular metabolite distributions suggests interplay of the cellular compartments for cysteine biosynthesis in Arabidopsis. Plant, Cell & Environment 32, 349–367. 10.1111/j.1365-3040.2008.01928.x 19143986

[CIT0034] Laureano-Marín AM, Moreno I, Romero LC, Gotor C 2016 Negative regulation of autophagy by sulfide is independent of reactive oxygen species. Plant Physiology 171, 1378–1391. 27208225 10.1104/pp.16.00110 PMC4902596

[CIT0035] Laxman S, Sutter BM, Wu X, Kumar S, Guo X, Trudgian DC, Mirzaei H, Tu BP 2013 Sulfur amino acids regulate translational capacity and metabolic homeostasis through modulation of tRNA thiolation. Cell 154, 416–429. 23870129 10.1016/j.cell.2013.06.043 PMC3757545

[CIT0036] Li L, Wang Y, Shen W 2012 Roles of hydrogen sulfide and nitric oxide in the alleviation of cadmium-induced oxidative damage in alfalfa seedling roots. BioMetals 25, 617–631. 22538639 10.1007/s10534-012-9551-9

[CIT0037] Li ZG, Gong M, Xie H, Yang L, Li J 2012 Hydrogen sulfide donor sodium hydrosulfide-induced heat tolerance in tobacco (Nicotiana tabacum L) suspension cultured cells and involvement of Ca(2+) and calmodulin. Plant Science 185-186, 185–189. 22325880 10.1016/j.plantsci.2011.10.006

[CIT0038] Ling J, Soll D 2010 Severe oxidative stress induces protein mistranslation through impairment of an aminoacyl-tRNA synthetase editing site. Proceedings of the National Academy of Sciences USA 107, 4028–4033. 10.1073/pnas.1000315107 PMC2840151 20160114

[CIT0039] Lisjak M, Srivastava N, Teklic T, Civale L, Lewandowski K, Wilson I, Wood ME, Whiteman M, Hancock JT 2010 A novel hydrogen sulfide donor causes stomatal opening and reduces nitric oxide accumulation. Plant Physiology and Biochemistry 48, 931–935. 20970349 10.1016/j.plaphy.2010.09.016

[CIT0040] Longen S, Richter F, Köhler Y, Wittig I, Beck KF, Pfeilschifter J 2016 Quantitative Persulfide Site Identification (qPerS-SID) reveals protein targets of h2s releasing donors in mammalian cells. Scientific Reports 6, 29808. 27411966 10.1038/srep29808 PMC4944133

[CIT0041] Lopez-Serra P, Marcilla M, Villanueva A 2014 A DERL3-associated defect in the degradation of SLC2A1 mediates the Warburg effect. Nature Communications 5, 3608. 10.1038/ncomms4608 PMC3988805 24699711

[CIT0042] Mishanina TV, Libiad M, Banerjee R 2015 Biogenesis of reactive sulfur species for signaling by hydrogen sulfide oxidation pathways. Nature Chemical Biology 11, 457–464. 26083070 10.1038/nchembio.1834 PMC4818113

[CIT0043] Miyakawa T, Fujita Y, Yamaguchi-Shinozaki K, Tanokura M 2013 Structure and function of abscisic acid receptors. Trends in Plant Science 18, 259–266. 23265948 10.1016/j.tplants.2012.11.002

[CIT0044] Mustafa AK, Gadalla MM, Sen N, Kim S, Mu W, Gazi SK, Barrow RK, Yang G, Wang R, Snyder SH 2009 H2S signals through protein S-sulfhydration. Science Signaling 2, ra72. 19903941 10.1126/scisignal.2000464 PMC2998899

[CIT0045] Oaks A 1994 Efficiency of nitrogen utilization in C3 and C4 cereals. Plant Physiology 106, 407–414. 12232337 10.1104/pp.106.2.407 PMC159544

[CIT0046] Olas B 2015 Hydrogen sulfide in signaling pathways. Clinica Chimica Acta 439, 212–218. 10.1016/j.cca.2014.10.037 25444740

[CIT0047] Pan J, Carroll KS 2013 Persulfide reactivity in the detection of protein s-sulfhydration. ACS Chemical Biology 8, 1110–1116. 23557648 10.1021/cb4001052 PMC3745995

[CIT0048] Papanatsiou M, Scuffi D, Blatt MR, García-Mata C 2015 Hydrogen sulfide regulates inward-rectifying K+ channels in conjunction with stomatal closure. Plant Physiology 168, 29–35. 25770153 10.1104/pp.114.256057 PMC4424018

[CIT0049] Park CM, Macinkovic I, Filipovic MR, Xian M 2015 Use of the “tag-switch” method for the detection of protein S-sulfhydration. Methods in Enzymology 555, 39–56. 25747474 10.1016/bs.mie.2014.11.033

[CIT0050] Park S, Imlay JA 2003 High levels of intracellular cysteine promote oxidative DNA damage by driving the fenton reaction. Journal of Bacteriology 185, 1942–1950. 12618458 10.1128/JB.185.6.1942-1950.2003 PMC150142

[CIT0051] Paul BD, Snyder SH 2012 H₂S signalling through protein sulfhydration and beyond. Nature Reviews. Molecular Cell Biology 13, 499–507. 22781905 10.1038/nrm3391

[CIT0052] Paul BD, Snyder SH 2015a H2S: A novel gasotransmitter that signals by sulfhydration. Trends in Biochemical Sciences 40, 687–700. 26439534 10.1016/j.tibs.2015.08.007 PMC4630104

[CIT0053] Paul BD, Snyder SH 2015b Modes of physiologic H2S signaling in the brain and peripheral tissues. Antioxidants & Redox Signaling 22, 411–423. 24684551 10.1089/ars.2014.5917 PMC4307159

[CIT0054] Paul BD, Snyder SH 2015c Protein sulfhydration. Methods in Enzymology 555, 79–90. 25747476 10.1016/bs.mie.2014.11.021

[CIT0055] Ramos-Fernández A, Paradela A, Navajas R, Albar JP 2008 Generalized method for probability-based peptide and protein identification from tandem mass spectrometry data and sequence database searching. Molecular & Cellular Proteomics 7, 1748–1754. 18515861 10.1074/mcp.M800122-MCP200 PMC2556015

[CIT0056] Riemenschneider A, Riedel K, Hoefgen R, Papenbrock J, Hesse H 2005 Impact of reduced O-acetylserine(thiol)lyase isoform contents on potato plant metabolism. Plant Physiology 137, 892–900. 15728339 10.1104/pp.104.057125 PMC1065390

[CIT0057] Romero LC, Aroca MÁ, Laureano-Marín AM, Moreno I, García I, Gotor C 2014 Cysteine and cysteine-related signaling pathways in Arabidopsis thaliana. Molecular Plant 7, 264–276. 24285094 10.1093/mp/sst168

[CIT0058] Romero LC, Aroca MÁ, Serna A, Gotor C 2013a Proteomic analysis of endogenous S-sulfhydration in Arabidopsis thaliana. Nitric Oxide 31, S23.

[CIT0059] Romero LC, García I, Gotor C 2013b L-Cysteine Desulfhydrase 1 modulates the generation of the signaling molecule sulfide in plant cytosol. Plant Signaling & Behavior 8, e24007. 23428891 10.4161/psb.24007 PMC3906162

[CIT0060] Rybak JN, Scheurer SB, Neri D, Elia G 2004 Purification of biotinylated proteins on streptavidin resin: a protocol for quantitative elution. Proteomics 4, 2296–2299. 15274123 10.1002/pmic.200300780

[CIT0061] Schmidt A, Trebst A 1969 The mechanism of photosynthetic sulfate reduction by isolated chloroplasts. Biochimica et Biophysica Acta 180, 529–535. 4390248 10.1016/0005-2728(69)90031-0

[CIT0062] Scuffi D, Álvarez C, Laspina N, Gotor C, Lamattina L, García-Mata C 2014 Hydrogen sulfide generated by L-cysteine desulfhydrase acts upstream of nitric oxide to modulate abscisic acid-dependent stomatal closure. Plant Physiology 166, 2065–2076. 25266633 10.1104/pp.114.245373 PMC4256879

[CIT0063] Shen J, Xing T, Yuan H, Liu Z, Jin Z, Zhang L, Pei Y 2013 Hydrogen sulfide improves drought tolerance in Arabidopsis thaliana by microRNA expressions. PLoS ONE 8, e77047. 24194857 10.1371/journal.pone.0077047 PMC3806758

[CIT0064] Shi H, Ye T, Chan Z 2013 Exogenous application of hydrogen sulfide donor sodium hydrosulfide enhanced multiple abiotic stress tolerance in bermudagrass (Cynodon dactylon (L). Pers.). Plant Physiology and Biochemistry 71, 226–234. 23974354 10.1016/j.plaphy.2013.07.021

[CIT0065] Sun J, Wang R, Zhang X, Yu Y, Zhao R, Li Z, Chen S 2013 Hydrogen sulfide alleviates cadmium toxicity through regulations of cadmium transport across the plasma and vacuolar membranes in Populus euphratica cells. Plant Physiology and Biochemistry 65, 67–74. 23416498 10.1016/j.plaphy.2013.01.003

[CIT0066] Takahashi H, Kopriva S, Giordano M, Saito K, Hell R 2011 Sulfur assimilation in photosynthetic organisms: molecular functions and regulations of transporters and assimilatory enzymes. Annual Review of Plant Biology 62, 157–184. 10.1146/annurev-arplant-042110-103921 21370978

[CIT0067] Thimm O, Bläsing O, Gibon Y 2004 MAPMAN: a user-driven tool to display genomics data sets onto diagrams of metabolic pathways and other biological processes. The Plant Journal 37, 914–939. 14996223 10.1111/j.1365-313x.2004.02016.x

[CIT0068] Van Hoewyk D, Pilon M, Pilon-Smits EAH 2008 The functions of NifS-like proteins in plant sulfur and selenium metabolism. Plant Science 174, 117–123.

[CIT0069] van Wijk KJ, Baginsky S 2011 Plastid proteomics in higher plants: current state and future goals. Plant Physiology 155, 1578–1588. 21350036 10.1104/pp.111.172932 PMC3091083

[CIT0070] Vandiver M, Snyder S 2012 Hydrogen sulfide: a gasotransmitter of clinical relevance. Journal of Molecular Medicine 90, 1–9. 22314625 10.1007/s00109-012-0873-4 PMC3901014

[CIT0071] Vescovi M, Zaffagnini M, Festa M, Trost P, Lo Schiavo F, Costa A 2013 Nuclear accumulation of cytosolic glyceraldehyde-3-phosphate dehydrogenase in cadmium-stressed Arabidopsis roots. Plant Physiology 162, 333–346. 23569110 10.1104/pp.113.215194 PMC3641213

[CIT0072] Vizcaíno JA, Csordas A, del-Toro N 2016 2016 update of the PRIDE database and its related tools. Nucleic Acids Research 44, D447–D456. 26527722 10.1093/nar/gkv1145 PMC4702828

[CIT0073] Wang BL, Shi L, Li YX, Zhang WH 2010 Boron toxicity is alleviated by hydrogen sulfide in cucumber (Cucumis sativus L.) seedlings. Planta 231, 1301–1309. 20224946 10.1007/s00425-010-1134-9

[CIT0074] Wang P, Du Y, Hou YJ, Zhao Y, Hsu CC, Yuan F, Zhu X, Tao WA, Song CP, Zhu JK 2015 Nitric oxide negatively regulates abscisic acid signaling in guard cells by S-nitrosylation of OST1. Proceedings of the National Academy of Sciences USA 112, 613–618. 10.1073/pnas.1423481112 PMC4299189 25550508

[CIT0075] Wang R 2014 Gasotransmitters: growing pains and joys. Trends in Biochemical Sciences 39, 227–232. 24767680 10.1016/j.tibs.2014.03.003

[CIT0076] Wedmann R, Onderka C, Wei S 2016 Improved tag-switch method reveals that thioredoxin acts as depersulfidase and controls the intracellular levels of protein persulfidation. Chemical Science 7, 3414–3426. 27170841 10.1039/c5sc04818d PMC4845716

[CIT0077] Yamaguchi Y, Nakamura T, Kusano T, Sano H 2000 Three Arabidopsis genes encoding proteins with differential activities for cysteine synthase and beta-cyanoalanine synthase. Plant & Cell Physiology 41, 465–476. 10845460 10.1093/pcp/41.4.465

[CIT0078] Zhang D, Macinkovic I, Devarie-Baez NO, Pan J, Park CM, Carroll KS, Filipovic MR, Xian M 2014 Detection of protein S-sulfhydration by a tag-switch technique. Angewandte Chemie 53, 575–581. 24288186 10.1002/anie.201305876 PMC4306352

[CIT0079] Zhang H, Hu LY, Hu KD, He YD, Wang SH, Luo JP 2008 Hydrogen sulfide promotes wheat seed germination and alleviates oxidative damage against copper stress. Journal of Integrative Plant Biology 50, 1518–1529. 19093970 10.1111/j.1744-7909.2008.00769.x

[CIT0080] Zhang H, Tan ZQ, Hu LY, Wang SH, Luo JP, Jones RL 2010 Hydrogen sulfide alleviates aluminum toxicity in germinating wheat seedlings. Journal of Integrative Plant Biology 52, 556–567. 20590986 10.1111/j.1744-7909.2010.00946.x

