# Supplementary material for: Persulfidation proteome reveals the regulation of protein function by hydrogen sulfide in diverse biological processes in Arabidopsis
Source: J Exp Bot. 2017 Aug 24;68(17):4915–27. doi: 10.1093/jxb/erx294 (PMC5853657; doi:10.1093/jxb/erx294)
Supplement: supplementary_figures_S1_S3 [file erx294_suppl_supplementary_figures_s1_s3.pdf]

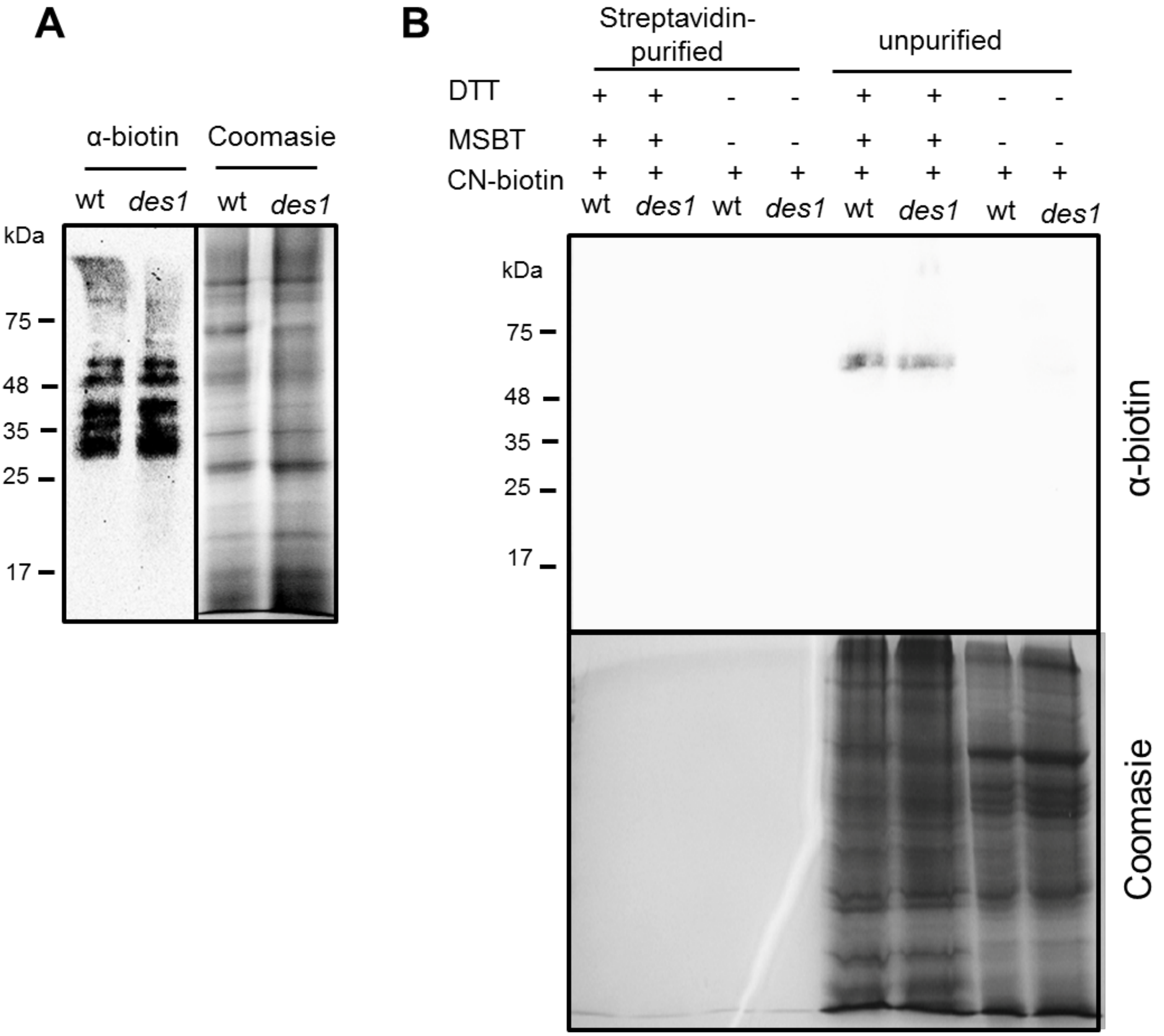

Supplemental Figure S1. Validation of the tag switch method in wild type and *des1* mutant Arabidopsis leaf extracts by immunoblotting with anti-biotin antibodies. (A) Western blot analysis of biotinylated proteins purified by the tag switch method from untreated leaf extracts. (B) Western blot analysis of biotinylated proteins from leaf extracts treated with 100 mM DTT prior to the blocking step with MSBT and the labeling step with CN-biotin, and for samples subjected only to the labeling step with CN-biotin. The right panel in B shows proteins analyzed after purification with streptavidin beads, and the left panel shows unpurified proteins. Coomassie blue staining is shown as a loading control.

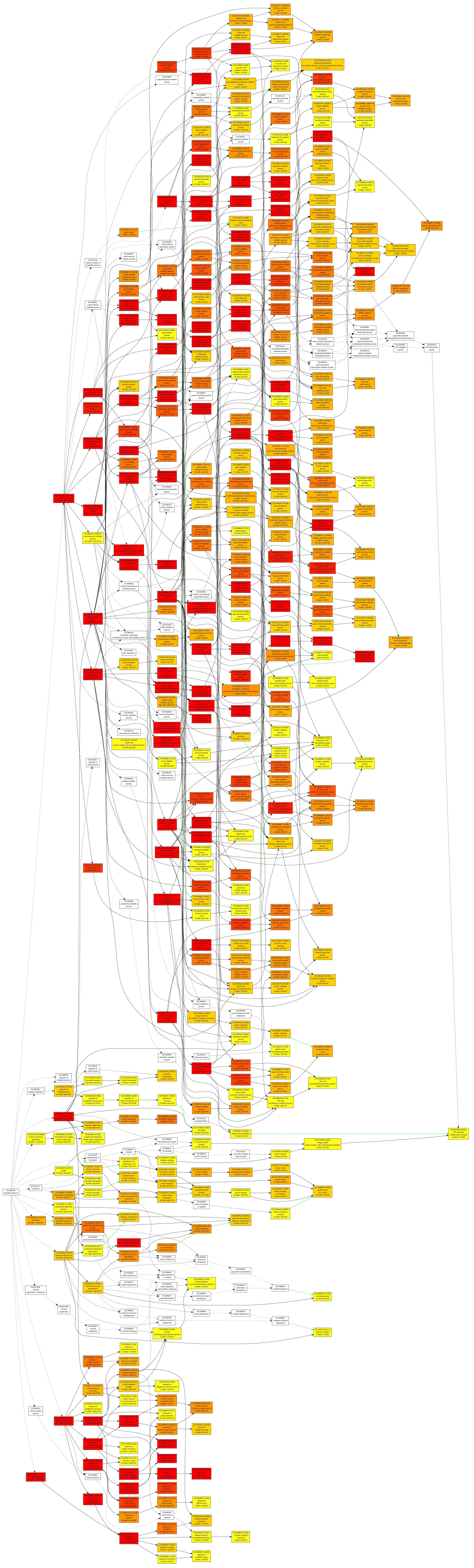

Supplemental Figure S2. Enriched Gene Ontology analysis of the 2,015 loci corresponding to the persulfidated proteins identified in wild type by the tag switch method. The graph was generated by Singular Enrichment Analysis (SEA) from AgriGO (Du et al, 2010). The graph displays the GO hierarchical image of the GO Biological Process categories containing statistically significant terms.

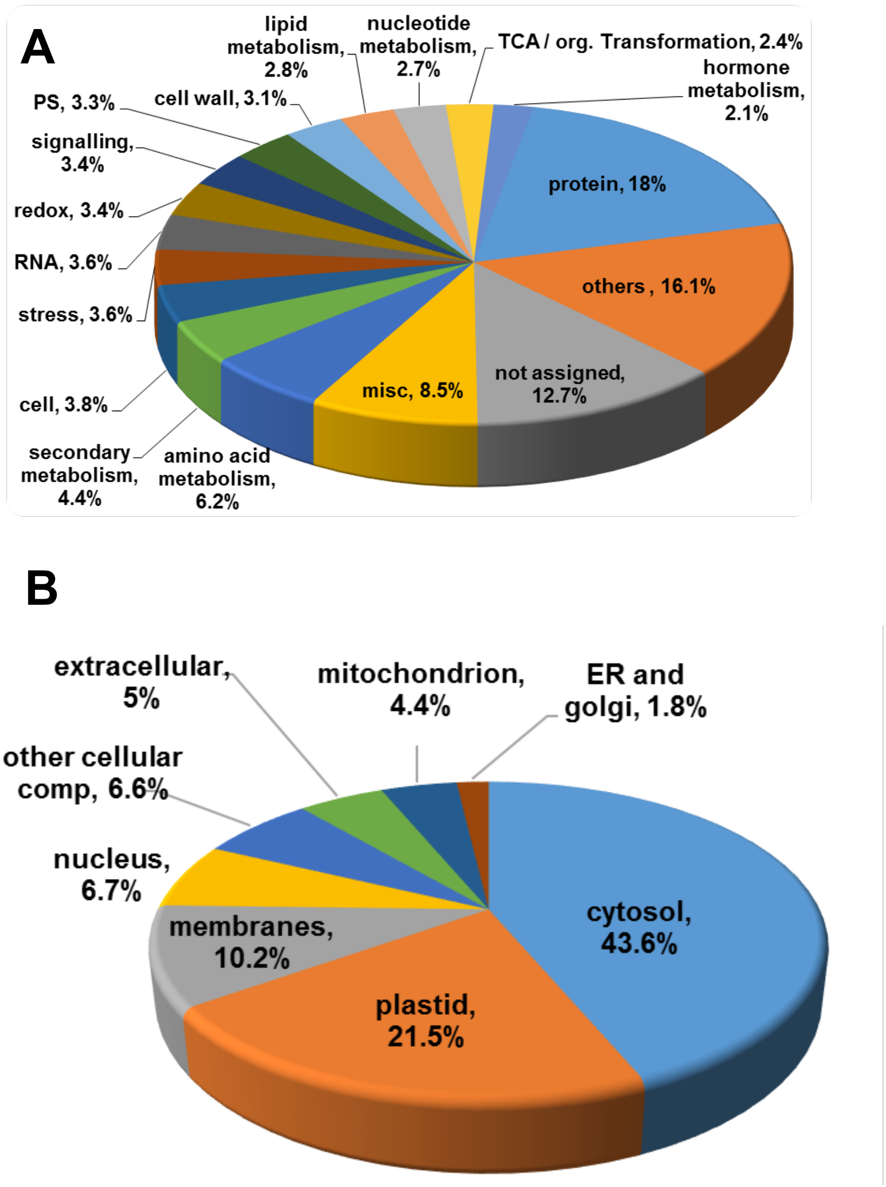

Supplemental Figure S3. Functional classification of S-sulfhydrated proteins identified by LC-MS/MS in the leaf extracts of 30-day-old Arabidopsis thaliana *des1* mutant plants. (A) Functional classification of gene ontology (GO) terms categorized by biological processes. (B) Functional classification by subcellular localization.
